# Transcriptional reference map of hormone responses in wheat spikes

**DOI:** 10.1186/s12864-019-5726-x

**Published:** 2019-05-20

**Authors:** Peng-Fei Qi, Yun-Feng Jiang, Zhen-Ru Guo, Qing Chen, Thérèse Ouellet, Lu-Juan Zong, Zhen-Zhen Wei, Yan Wang, Ya-Zhou Zhang, Bin-Jie Xu, Li Kong, Mei Deng, Ji-Rui Wang, Guo-Yue Chen, Qian-Tao Jiang, Xiu-Jin Lan, Wei Li, Yu-Ming Wei, You-Liang Zheng

**Affiliations:** 1 State Key Laboratory of Crop Genetics of Disease Resistance and Disease Control, Chengdu, 611130 Sichuan China; 2 Triticeae Research Institute, Sichuan Agricultural University, Chengdu, 611130 Sichuan China; 3 Ottawa Research and Development Centre, Agriculture and Agri-Food Canada, 960 Carling Avenue, Ottawa, ON K1A 0C6 Canada

**Keywords:** Phytohormone, Transcriptome, Resistance, Fusarium head blight, Water deficit, Marker gene, Defence mechanism

## Abstract

**Background:**

Phytohormones are key regulators of plant growth, development, and signalling networks involved in responses to diverse biotic and abiotic stresses. Transcriptional reference maps of hormone responses have been reported for several model plant species such as *Arabidopsis thaliana*, *Oryza sativa*, and *Brachypodium distachyon.* However, because of species differences and the complexity of the wheat genome, these transcriptome data are not appropriate reference material for wheat studies.

**Results:**

We comprehensively analysed the transcriptomic responses in wheat spikes to seven phytohormones, including indole acetic acid (IAA), gibberellic acid (GA), abscisic acid (ABA), ethylene (ET), cytokinin (CK), salicylic acid (SA), and methyl jasmonic acid (MeJA). A total of 3386 genes were differentially expressed at 24 h after the hormone treatments. Furthermore, 22.7% of these genes exhibited overlapping transcriptional responses for at least two hormones, implying there is crosstalk among phytohormones. We subsequently identified genes with expression levels that were significantly and differentially induced by a specific phytohormone (i.e., hormone-specific responses). The data for these hormone-responsive genes were then compared with the transcriptome data for wheat spikes exposed to biotic (Fusarium head blight) and abiotic (water deficit) stresses.

**Conclusion:**

Our data were used to develop a transcriptional reference map of hormone responses in wheat spikes.

**Electronic supplementary material:**

The online version of this article (10.1186/s12864-019-5726-x) contains supplementary material, which is available to authorized users.

## Background

Common wheat (*Triticum aestivum*) is one of the most important cereal crops worldwide because of its production and use. Additionally, because of its unique processing quality, wheat is consumed in many specific forms, including as bread or steamed bread, pizza, noodles, cake, biscuits, and dumplings. Common wheat is a hexaploid species (2n = 6x = 42), with a large genome comprising 16 gigabases [1]. The complexity of the wheat genome has impeded the elucidation of the roles of phytohormones in wheat plants, in contrast to model plant species.

Phytohormones are key molecules for regulating plant growth, development, and signalling networks involved in responses to diverse biotic and abiotic stresses [2–4]. Moreover, they function as part of a complex network that finely regulates gene expression in response to environmental cues. The biosynthesis, catabolism, transport, and signalling pathways of the major hormones [i.e., auxin, gibberellin (GA), abscisic acid (ABA), cytokinin (CK), ethylene (ET), salicylic acid (SA), and jasmonic acid (JA)] have been widely investigated in model plant species such as *Arabidopsis thaliana* and rice [5–10]. Phytohormones affect wheat yield, pre-harvest sprouting, and Fusarium head blight (FHB) resistance, all of which are related to spikes. The global wheat yield has substantially increased since the 1960s largely because of the Green Revolution [11], with one of the wheat Green Revolution genes encoding a mutant GA-responsive protein, DELLA [12]. Auxins can increase the final wheat harvest [13]. Additionally, GA, ABA, and auxins (IAA) regulate pre-harvest sprouting and seed dormancy [14–16]. Both wheat yield and quality are negatively affected by FHB, which is a devastating disease worldwide [17]. Infections by *Fusarium graminearum* (i.e., the major causal agent of FHB) lead to altered endogenous phytohormone levels in the wheat spikes [18]. The effects of JA, ABA, IAA, ET, and SA on FHB have been reported [18–24]. Moreover, *F. graminearum* and JA and/or ABA treatments have a synergistic effect on the expression of *ExpB6* (β-expansin 6), *Pdf1.2* (plant defensin 1.2), and *PR4* (pathogenesis-related protein 4). Furthermore, *F. graminearum* and JA treatments have an antagonistic effect on *ATB2* (auxin-inducible oxidoreductase) expression [18].

The transcriptome refers to the total mRNA content in an organism or in a specific type of tissue or cell. A transcriptome analysis enables researchers to characterise the global transcriptional activity and to identify a subset of target genes relatively easily. Considering the importance of phytohormones, transcriptome analyses are essential for elucidating the key roles of phytohormones, and have been conducted for several model plant species such as *A. thaliana* (135 megabases) [10], rice (389 megabases) [25], and *Brachypodium distachyon* (272 megabases) [26]. The resulting comprehensive transcriptome data have been widely used in other studies regarding the largescale or gene-specific regulation of transcripts. However, these transcriptome data cannot be used as reference material for studies on wheat spike-related traits because of species differences and the complexity of the common wheat genome (16 gigabases). Nevertheless, transcriptome analyses are still widely used for the global and rapid identification of differentially expressed genes (DEGs) under various conditions. Therefore, the phytohormone-regulated transcriptomic changes in wheat spikes can and should be analysed.

In this study, we completed a comprehensive analysis of the transcriptomic changes in wheat spikes in response to seven phytohormones [IAA, GA (GA_3_), ABA, ET, CK (trans-zeatin), SA, and MeJA] to identify responsive genes, investigate the crosstalk among hormones, and develop quantitative real-time polymerase chain reaction (qRT-PCR) markers for hormone signalling. The resulting data were then used for combined analyses of the transcriptomic changes due to biotic (FHB) and abiotic (water deficit) stresses. The results presented herein may be useful for clarifying the effects of phytohormones on wheat spike-related traits.

## Results

### Identification of differentially expressed genes

**Fig. 1:**
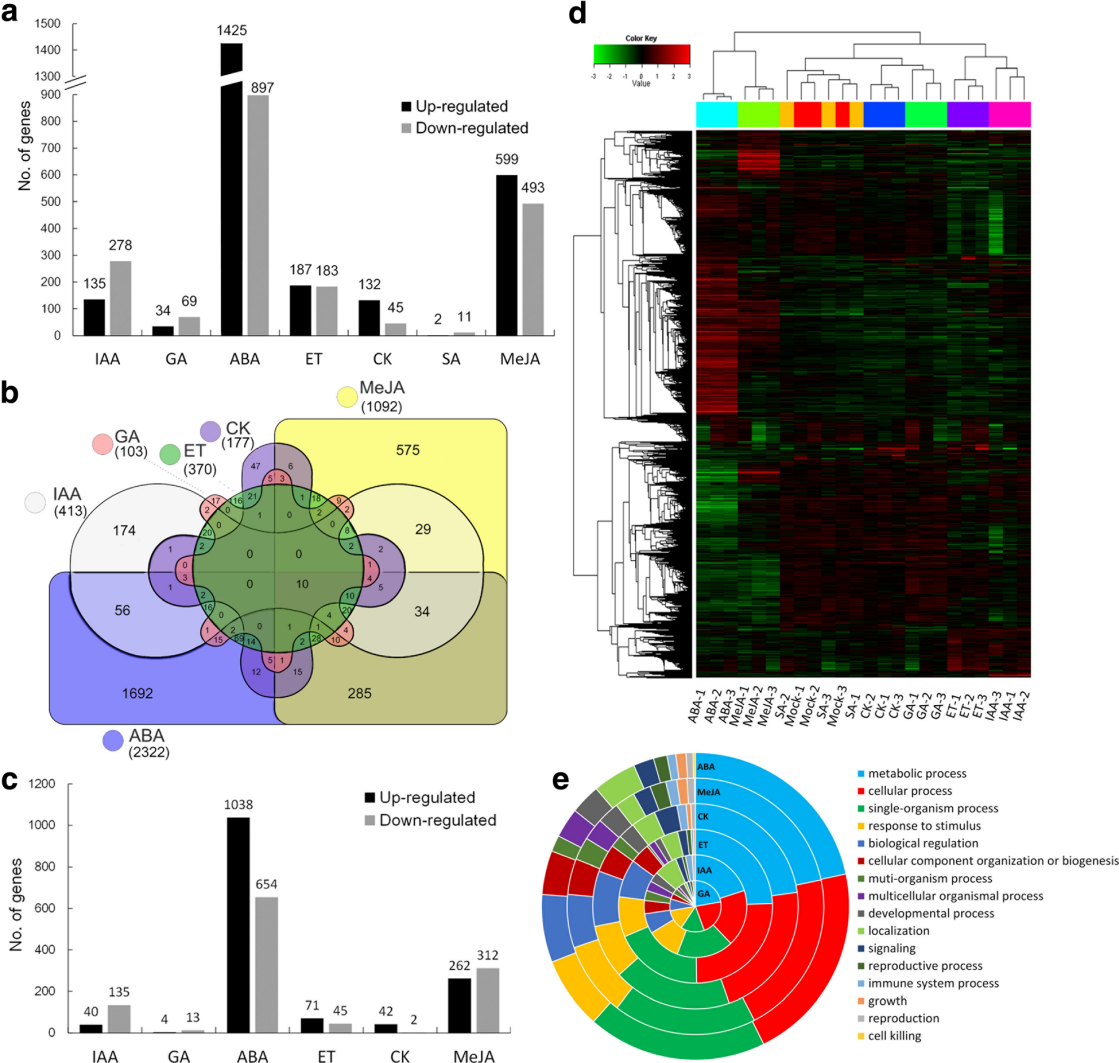
Differentially expressed genes (DEGs) in hormone-treated wheat spikes. **a** Number of DEGs regulated by various plant hormones. **b** Overlapping and unique DEGs in response to various plant hormones (excluding SA). **c** Number of unique DEGs regulated by various plant hormones. **d** Heat map illustrating the hierarchical clustering results for the microarray data. Mock, water treatment. **e** Gene ontology classification of DEGs

Microarray data revealed gene expression changes in wheat spikes in response to all seven tested hormones. A total of 3386 DEGs were identified (Additional file 1: Table S1). The application of exogenous phytohormones upregulated gene expression levels as follows: 135 genes for IAA, 34 genes for GA_3_, 1425 genes for ABA, 187 genes for ET, 132 genes for trans-zeatin, 2 genes for SA, and 599 genes for MeJA (Fig. 1a). The phytohormone treatments downregulated gene expression levels as follows: 278 genes for IAA, 69 genes for GA_3_, 897 genes for ABA, 183 genes for ET, 45 genes for trans-zeatin, 11 genes for SA, and 493 genes for MeJA (Fig. 1a). Moreover, ABA and SA exhibited the strongest and weakest effects on transcript abundance, respectively.

Among the identified DEGs, only one (Ta.12812) was common to all seven phytohormones. We observed some overlapping gene expression, but the expression levels of many genes were uniquely upregulated or downregulated by individual hormones (Fig. 1b). Specifically, the expression levels of 40, 4, 1038, 71, 42, and 262 genes were uniquely upregulated by IAA, GA, ABA, ET, trans-zeatin, and MeJA, respectively, whereas the expression levels of 135, 13, 654, 45, 2, and 312 genes were uniquely downregulated by IAA, GA, ABA, ET, trans-zeatin, and MeJA, respectively (Fig. 1c). We did not detect any gene uniquely regulated by SA under our experimental conditions.

A hierarchical clustering analysis to compare global gene expression changes (Fig. 1d) indicated that the differences between the drought stress and hormone treatments (except SA) were significant. The global expression patterns induced by MeJA, GA, and ET were similar to those induced by ABA, CK, and IAA, respectively.

### Gene ontology classification of DEGs affected by phytohormones

A gene ontology (GO) classification of DEGs was completed to identify biological processes affected by six of the seven hormone treatments (the exception was SA) (Fig. 1e). Genes associated with the GO terms ‘cellular processes’, ‘single-organism processes’, and ‘metabolic processes’ were generally the most affected by the hormone treatments. Notably, genes associated with the GO term ‘response to stimulus’ were strongly affected by the six hormone treatments. Significant GO terms were identified for each of the six hormone treatments (Additional file 1: Table S2). Additionally, the gene response patterns varied among the hormone treatments.

### Antagonistic and synergistic interactions between phytohormones

**Fig. 2:**
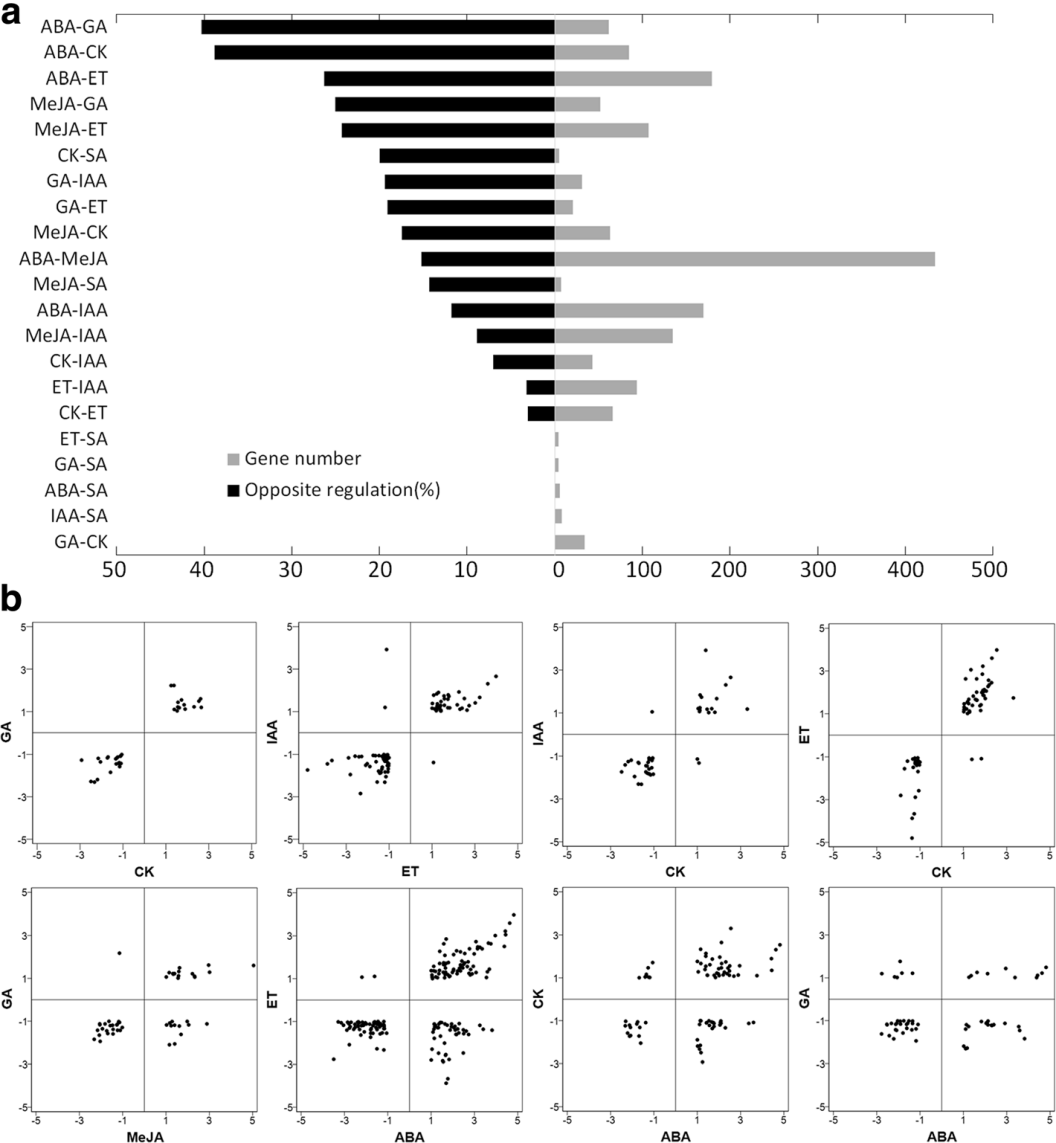
Antagonistic and synergistic interactions between phytohormones. **a** Percentage of antagonistically regulated genes between pairs of hormones. Gene number refers to the number of overlapping genes regulated by the two corresponding hormones. **b** Scatter plots of fold-changes in the overlapping genes between pairs of hormones. The vertical and horizontal ordinates indicate expression values presented in the log_2_-transformed form

In this study, 22.7% of the DEGs were regulated by two or more hormones. These overlapping genes might be important for the crosstalk among hormones. The percentage of antagonistically regulated genes obviously varied between different hormone pairs (Fig. 2a). In particular, almost no opposite responses were identified for the overlapping genes between GA and CK, IAA and ET, IAA and CK, and ET and CK (Fig. 2b), indicating extensive synergy between these hormones at the transcriptional level. In contrast, there were considerable opposite responses for the overlapping genes between GA and MeJA, ABA and ET, CK and ABA, and GA and ABA (Fig. 2b).

### Validation of gene expression by qRT-PCR

**Fig. 3:**
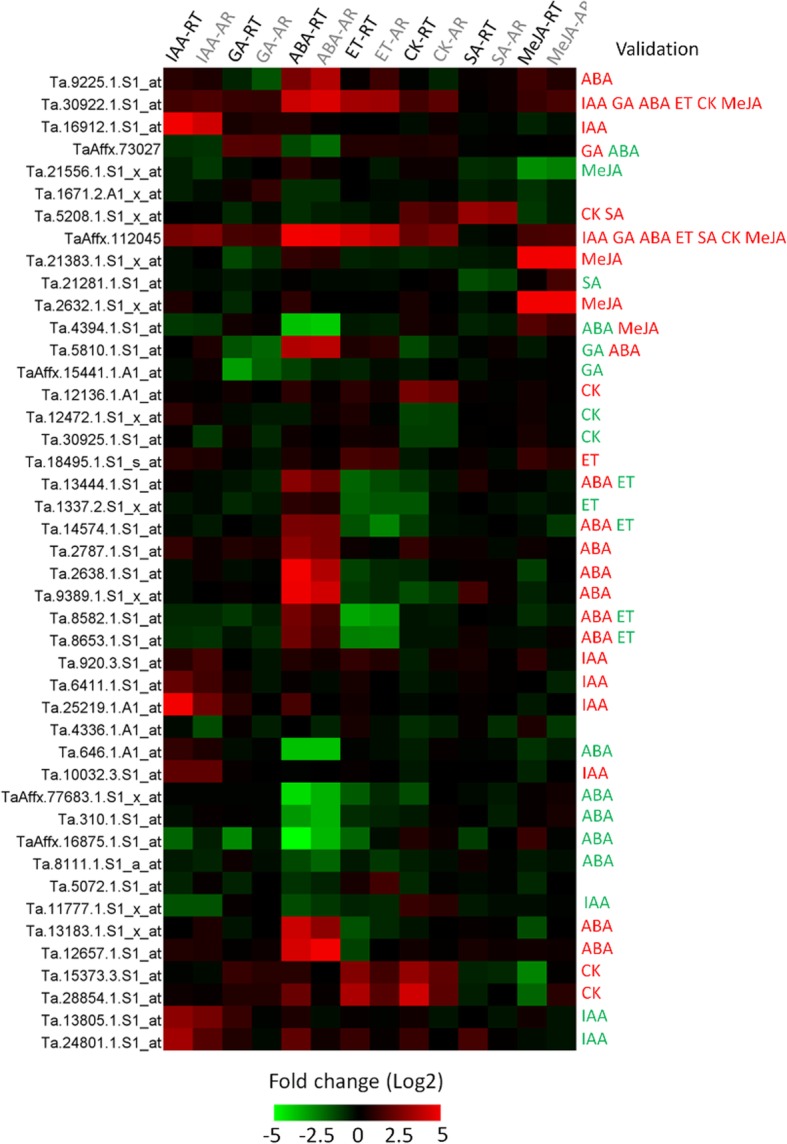
Validation of the expression of 44 genes by qRT-PCR. Expression-level changes based on qRT-PCR and microarray data are indicated by RT and AR, respectively. Significant gene expression changes validated by qRT-PCR in response to hormones are listed on the right side. Two or more hormones in one line indicate overlapping results. Red and green refer to upregulated and downregulated expression, respectively

Microarray data were verified by the qRT-PCR analysis of 44 DEGs (Additional file 1: Table S1). The expression patterns of the 44 genes as determined by qRT-PCR were largely consistent with those obtained from the microarray analysis (Fig. 3).

Some of the genes were identified as suitable hormone response markers in wheat spikes because they were significantly and differentially regulated by a specific phytohormone. A gene specifically induced by SA (Ta.5208.1.S1_x_at, which encodes a thionin-like protein) exhibited approximately 6.9-fold and 8.5-fold increases in expression levels in response to SA according to microarray and qRT-PCR analyses, respectively. Similarly, the expression levels of genes encoding an auxin-responsive protein (Ta.16912.1.S1_at), a seed maturation protein (Ta.9389.1.S1_x_at), a flavonol 3-sulfotransferase (Ta.12136.1.A1_at), and a chymotrypsin inhibitor (Ta.2632.1.S1_x_at) were upregulated by IAA, ABA, CK, and MeJA, respectively, according to the microarray and qRT-PCR data. Conversely, the expression of TaAffx.15441.1.A1_at, Ta.646.1.A1_at, Ta.8582.1.S1_at, and Ta.21556.1.S1_x_at was inhibited by GA, ABA, ET, and MeJA, respectively.

### Expression of hormone-responsive genes during an *F. graminearum* infection

**Fig. 4:**
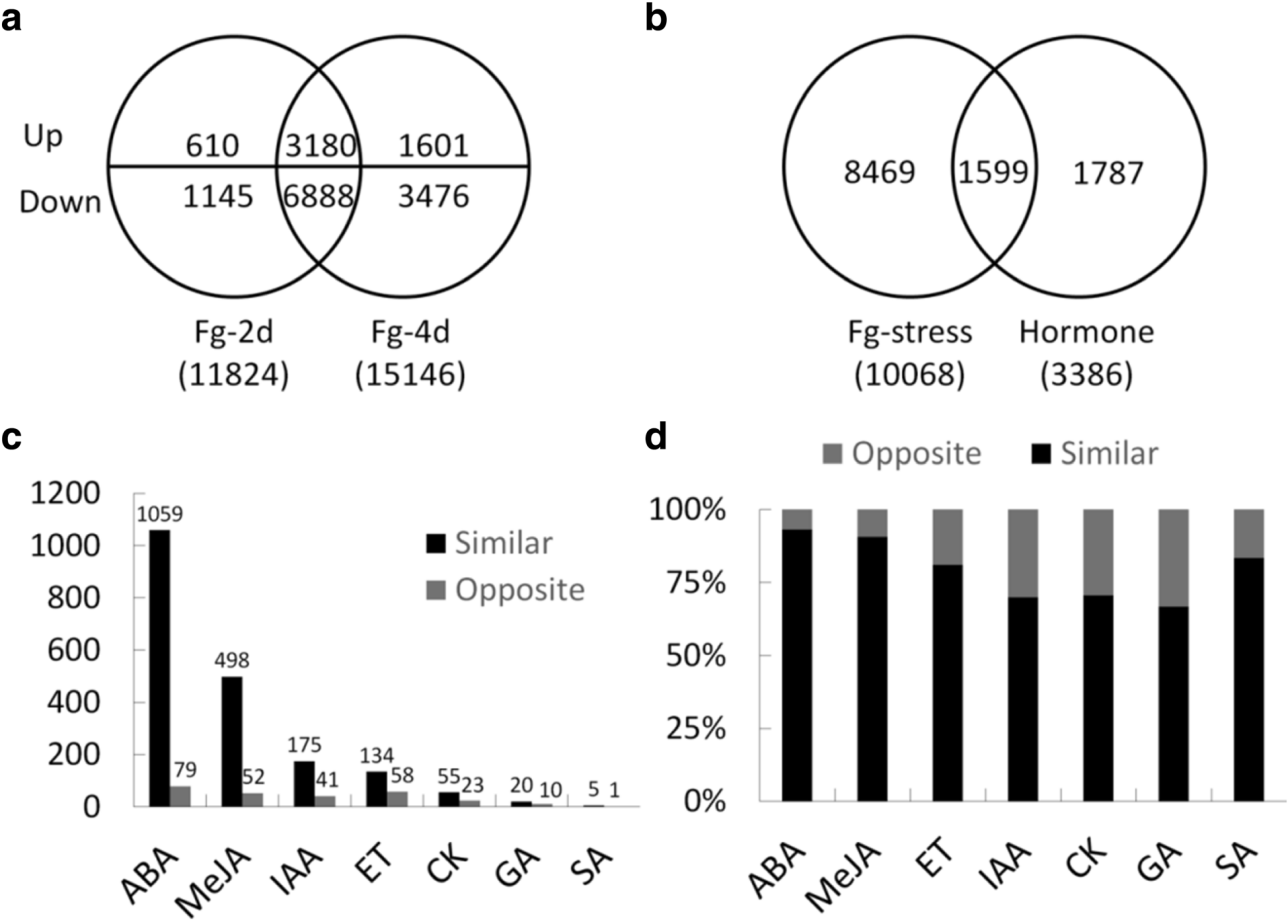
Expression of hormone-responsive genes during an *F. graminearum* infection. **a** Number of upregulated and downregulated genes in *T. aestivum* cv. ‘Roblin’ spikes at 2 (Fg-2d) and 4 days (Fg-4d) post-inoculation. Specific and common genes were categorised from the non-intersecting and intersecting areas, respectively. Up, upregulated genes; Down, downregulated genes. **b** Venn diagrams presenting the genes responsive to *F. graminearum* (Fg-stress) and hormone treatments. **c** Number of differentially expressed genes (DEGs) exhibiting similar or the opposite expression patterns in spike samples treated with *F. graminearum* or hormones. **d** Percentage of DEGs exhibiting similar or the opposite expression patterns in spike samples treated with *F. graminearum* or hormones

To better characterise the phytohormone functions related to wheat resistance against *F. graminearum*, the transcriptome data for *T. aestivum* cv. ‘Roblin’ plants infected with FHB (GEO record #GSE54554) were compared with our data. A total of 10,068 DEGs, including 3180 upregulated and 6888 downregulated genes, were identified at 2 and 4 days post-inoculation with *F. graminearum* (Fig. 4a). Moreover, 1599 of the 10,086 DEGs were responsive to both the *F. graminearum* infection and hormone treatments (Fig. 4b).

#### SA

Six DEGs were responsive to SA (Fig. 4c; Additional file 1: Table S3), and five of them, including Ta.5208.1.S1_x_at (i.e., SA-response marker gene) were similarly expressed following an *F. graminearum* infection (fungal stress) and SA treatment.

#### MeJA

A total of 550 DEGs were identified as MeJA-responsive genes, and 90.55% (498/550) of these DEGs exhibited similar expression patterns in response to fungal stress and MeJA (Fig. 4c and d; Additional file 1: Table S3). A GO enrichment analysis indicated that multiple defence-related processes were over-represented, including glutathione conjugation reactions and metabolic processes, sulfur metabolic processes, responses to oxidative stress, heterocycle biosynthetic processes, and peroxidase reactions (Additional file 1: Table S4). Excluding the interference of other hormones, 273 DEGs were specifically regulated by MeJA, 85 of which exhibited upregulated expression in response to both fungal stress and MeJA. An examination of the putative functions of these 85 genes revealed that they are involved in DON detoxification, phenylpropanoid pathways for secondary cell wall thickening, peroxidase reactions, GDSL-lipase reactions, cell wall defence, and defence signalling (Table 1).

**Table 1:** Defence-related genes specifically upregulated by MeJA and *F. graminearum*

Probe Set	Annotation	Flod change (log2)		
		Fg-2d	Fg-4d	MeJA
DON detoxification				
Ta.12808.1.S1_at	PDR-like ABC transporter	18.06	16.20	2.39
Ta.21281.2.A1_at	similar to PDR-like ABC transporter	8.62	11.66	2.44
TaAffx.1140.1.A1_at	glutathione S-transferase	7.42	15.43	3.35
Ta.13496.1.A1_at	UDP-glucosyltransferase	3.24	4.13	2.21
Ta.22589.1.S1_at	UDP-glucosyltransferase	3.58	6.05	2.18
Ta.18630.1.A1_at	cytochrome P450	10.84	13.91	2.25
Ta.18630.1.A1_x_at	cytochrome P450	10.83	10.57	2.30
Ta.1875.1.S1_at	cytochrome P450	10.18	51.10	2.40
Ta.1875.2.S1_at	cytochrome P450	9.45	26.90	2.56
Ta.1875.2.S1_x_at	cytochrome P450	10.05	34.93	2.37
TaAffx.105598.1.S1_at	cytochrome P450	4.52	2.06	2.37
Secondary cell wall				
Ta.8618.1.S1_at	shikimate kinase	47.61	59.14	2.17
Ta.9122.1.S1_at	arogenate dehydratase 1	13.86	28.33	2.72
Ta.9122.1.S1_x_at	arogenate dehydratase 1	14.02	25.07	2.86
Ta.9122.2.S1_at	arogenate dehydratase 1	5.91	4.88	2.31
Ta.16968.1.A1_at	4-coumarate--CoA ligase	15.38	14.90	2.48
Ta.8228.1.S1_at	agmatine coumaroyltransferase	22.31	83.23	8.60
TaAffx.109981.1.S1_x_at	agmatine coumaroyltransferase	12.31	7.71	4.13
TaAffx.29050.1.S1_s_at	agmatine coumaroyltransferase	24.29	33.47	4.80
Ta.14545.1.S1_at	O-methyltransferase	267.12	205.33	2.99
Peroxidase				
Ta.18497.1.S1_at	Peroxidase	23.37	10.47	2.31
Ta.21505.1.S1_at	peroxidase	15.31	7.12	2.03
Ta.24106.1.S1_x_at	peroxidase	8.29	2.59	3.94
Ta.24710.1.S1_at	Peroxidase	6.01	2.46	4.13
TaAffx.39568.2.S1_at	Peroxidase	4.26	2.53	5.04
Defense genes				
Ta.14766.1.S1_at	NBS-LRR type disease resistance protein RPG1-B	17.96	5.14	2.28
GDSL-lipases				
Ta.5520.1.S1_at	GDSL lipase	3.40	2.36	2.06
Cell wall defence				
Ta.21262.1.A1_at	xylanase inhibitor precursor	16.40	23.60	2.45
Ta.21262.1.A1_x_at	xylanase inhibitor precursor	16.13	23.60	2.54
Ta.19591.2.A1_a_at	Glucan 1,3-beta-glucosidase precursor	2.88	3.78	2.38
Transcription and signalling				
Ta.4678.1.S1_at	WRKY transcription factor	9.67	31.06	2.31
Ta.4678.1.S1_x_at	WRKY transcription factor	8.87	29.85	2.28
Ta.30507.2.S1_x_at	ZIM domain containing protein	13.60	20.22	2.11
Ta.30507.1.S1_a_at	ZIM domain containing protein	12.70	18.32	2.30
Ta.9507.2.S1_x_at	zinc-finger protein	35.96	7.822	2.78
TaAffx.120360.1.A1_at	similar to heat shock transcription factor	15.24	33.62	3.34
TaAffx.76510.1.S1_at	similar to MADS-box protein FDRMADS	3.45	2.60	2.20

#### ABA

We identified 1138 DEGs as ABA-responsive genes, and 93.06% (1059/1138) of these genes were similarly expressed after fungal stress and ABA treatments (Fig. 4c and d; Additional file 1: Table S3). Among these DEGs, 777 were specifically regulated by ABA, and 92.41% (718/777) of these DEGs produced similar expression patterns in response to fungal stress and ABA treatments. We previously confirmed that JA and ABA differentially regulate wheat resistance against *F. graminearum*, with JA significantly enhancing resistance and ABA having the opposite effect [18]. Unexpectedly, multiple defence processes were over-represented among the DEGs upregulated by ABA, including glutathione conjugation reactions and metabolic processes, cell wall polysaccharide metabolic processes, xylan catabolic processes, and sulfur metabolic processes (Additional file 1: Table S4). In contrast, phenylpropanoid metabolic and biosynthetic processes were enriched among the DEGs downregulated by ABA. Indeed, the expression levels of multiple types of phenylpropanoid pathway genes involved in lignin and flavonoid biosynthesis were downregulated by ABA, including genes encoding caffeic acid-O-methyltransferase, dihydroflavonol-4-reductase, flavonoid 3′-monooxygenase, chalcone synthase, chitinase, and dirigent proteins (Table 2). Obviously, ABA can promote *F. graminearum* infections in wheat by inhibiting the biosynthetic processes related to the plant secondary cell wall, even though ABA also upregulates the expression of many genes associated with resistance against *F. graminearum*.

**Table 2:** Phenylpropanoid pathway genes downregulated by ABA and *F. graminearum*

Probe set	Category	Annotation	Fold change (log2)		
			ABA	Fg-2d	Fg-4d
TaAffx.115378.1.S1_at	Phenylpropanoid biosynthesis	caffeic acid 3-O-methyltransferase	0.42	0.07	0.12
Ta.9172.1.S1_x_at	flavonoid biosynthetic process	chalcone synthase	0.25	0.07	0.13
Ta.9172.2.S1_x_at	flavonoid biosynthetic process	chalcone synthase	0.41	0.28	0.21
Ta.9172.3.S1_x_at	flavonoid biosynthetic process	chalcone synthase	0.32	0.29	0.18
Ta.12690.2.S1_x_at	flavonoid biosynthetic process	dihydroflavonol-4-reductase	0.44	0.30	0.28
TaAffx.37978.1.A1_at	flavonoid biosynthetic process	Flavonoid 3′-monooxygenase	0.31	0.39	0.32
Ta.4385.2.A1_at	Lignin Biosynthesis	chitinase-like protein 2	0.43	0.22	0.15
Ta.4455.1.A1_at	lignin Biosynthesis	laccase - like protein	0.36	0.17	0.14
Ta.25384.1.S1_at	Lignin Biosynthesis	dirigent-like protein	0.38	0.45	0.31
TaAffx.132123.1.A1_x_at	Lignin Biosynthesis	dirigent protein	0.22	0.28	0.19

#### IAA

A total of 216 IAA-responsive DEGs were detected, of which 81.02% (175/216) were similarly expressed following fungal stress and IAA treatments (Fig. 4c and d). Similar to the effects of MeJA and ABA, the expression levels of many defence-related genes were upregulated by IAA, but 75.79% (72/95) of the upregulated genes were not specifically regulated by IAA.

#### ET

We determined that 192 DEGs were responsive to ET, and 69.79% (134/192) of these DEGs exhibited similar expression patterns in response to fungal stress and ET (Fig. 4c and d). Only 40 DEGs were specifically regulated by ET, of which 23 were similarly expressed during exposures to fungal stress and exogenous ET.

#### CK and GA

Among the detected DEGs, 78 and 30 were responsive to CK and GA, respectively (Fig. 4c; Additional file 1: Table S3). Additionally, 70.51% (55/78) and 66.67% (20/30) of these DEGs produced similar expression patterns in response to fungal stress and the corresponding hormone, respectively (Fig. 4d). Only 8 and 5 of these DEGs were specifically regulated by CK and GA, respectively.

### Expression of hormone-responsive genes in the wheat glume under water deficit conditions

**Fig. 5:**
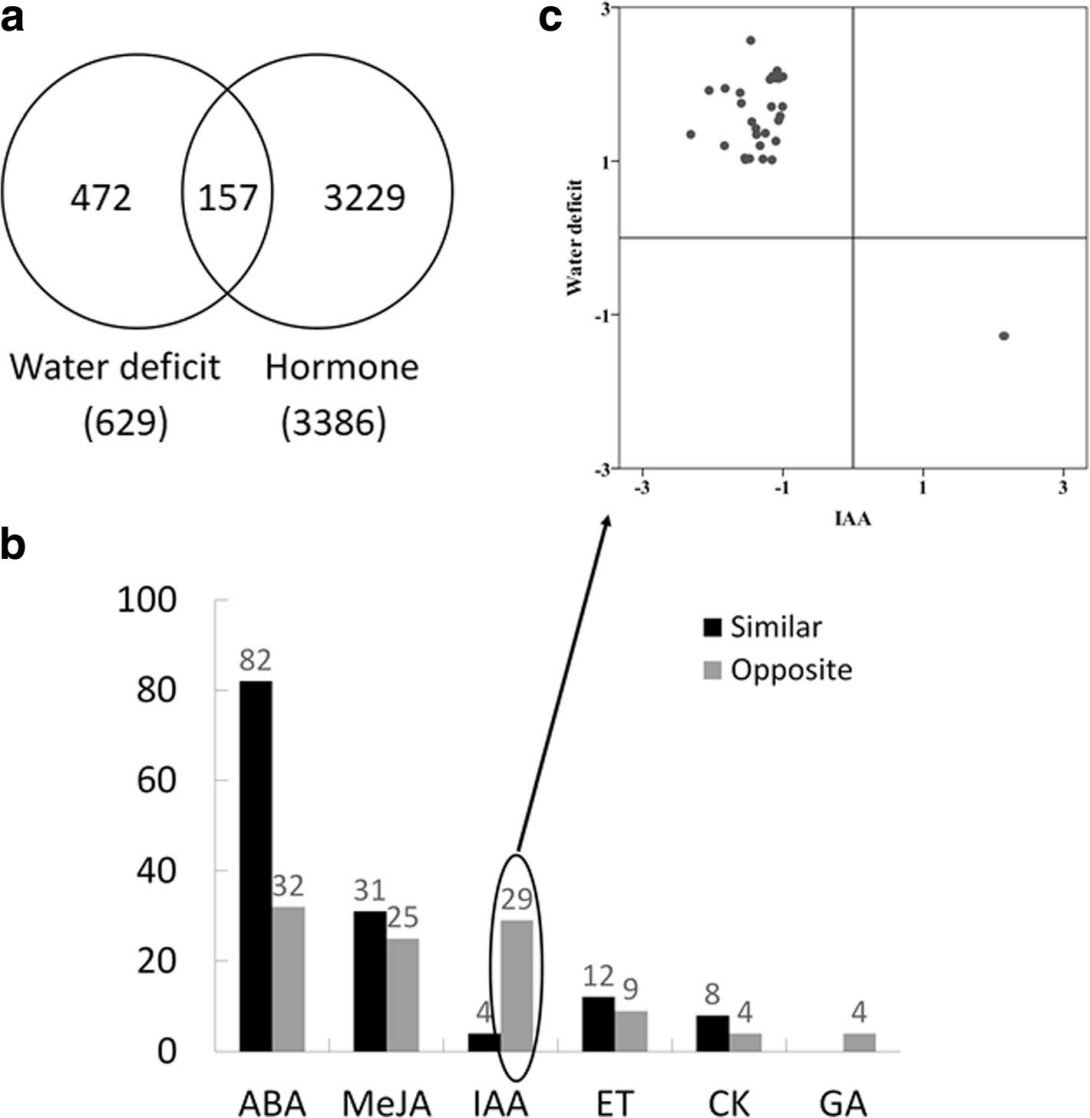
Expression of hormone-responsive genes in the wheat glume in response to water deficit stress. **a** Venn diagrams presenting the genes responsive to water deficit stress and hormone treatments. **b** Number of differentially expressed genes exhibiting similar and the opposite expression patterns in spike samples treated with water deficit stress or hormones. **c** Scatter plots of the fold-changes for the 29 genes that exhibited the opposite expression patterns in response to water deficit stress and an IAA treatment. The vertical and horizontal ordinates indicate the expression values presented in the log_2_-transformed form

Wheat glume, which is the main green tissue of spikes, facilitates photosynthesis and delays aging under drought conditions [27]. To clarify the effects of the major hormones in the wheat glume in response to drought stress, we compared the available data for 629 DEGs under water deficit conditions at 6 days after anthesis [28] with the data generated in the current study. Of these 629 DEGs, 157 were identified as hormone-responsive genes (Fig. 5a; Additional file 1: Table S5).

We determined that 71.92% (82/114) of the ABA-responsive genes (Fig. 5b) were similarly expressed in response to ABA and drought stress, whereas 87.88% (29/33) of the IAA-responsive genes (Fig. 5b; Additional file 1: Table S6) exhibited the opposite expression patterns following IAA and water deficit treatments. Moreover, the expression levels of almost all of these IAA-responsive genes (28/29) were downregulated by IAA, but were upregulated by drought stress (Fig. 5c). These results suggested that the drought resistance of the wheat glume may be improved by inhibiting IAA signalling. Functional annotations further implied that these 29 genes contribute to the drought resistance of the wheat glume by encoding the vesicle-associated membrane protein, serine/threonine protein kinase, receptor-like protein kinase, cytochrome P450, xyloglucan endotransglucosylase/hydrolase protein, lipoxygenase, and the cold acclimation protein (Additional file 1: Table S6).

## Discussion

Comprehensive transcriptome analyses following hormone treatments have been completed for model plant species, including *A. thaliana* [10], rice [25], and *B. distachyon* [26]; however, these studies focused on the seedling stage. Gene expression patterns differ dramatically between organs and tissues because of a temporally and spatially regulated process involving the selective expression of specific parts of the genome [29, 30]. Common wheat, which is an allohexaploid species comprising three genomes (A, B, and D), has multiple orthologous genes. The expression of these genes for coordinated responses to diverse stimuli involves a very complex mechanism [31, 32]. Additionally, the complexity of the wheat genome contributes to the difficulties associated with analyses using transcriptional data from diploid species. We previously determined that caution should be exercised when using traditional *A. thaliana* marker genes to investigate wheat [20]. In the current study, we comprehensively analysed the transcriptomic changes in wheat spikes induced by seven phytohormones. A comparison between the transcriptome data for *B. distachyon* and rice [25, 26] and the data generated in this study indicated that only a few genes exhibited the same expression pattern in wheat spikes (data not shown), highlighting the importance of the transcriptional reference map of hormone responses in wheat spikes.

Satisfying the growing demand for wheat worldwide has been challenging [33]. Research on the effects of phytohormones may contribute to increased productivity to narrow the gap between the demand and supply. Since the 1960s, the global wheat yield has substantially increased as a result of the the manipulation of GA signaling [11, 12]. Other GA-responsive dwarfism genes, such as *Rht4*, *Rht5*, *Rht8*, *Rht12*, and *Rht13*, have the potential to increase bread wheat yield without compromising aerial biomass or coleoptile length. The use of uniconazole, a plant growth regulator that inhibits GA biosynthesis, also enhances wheat production [34]. Additionally, an earlier investigation proved that increasing the auxin level positively influences the final wheat yield [13]. In rice, *OsCKX2* encodes a cytokinin oxidase/dehydrogenase, which degrades CK. Thus, downregulated *OsCKX2* expression results in the accumulation of CK in rice inflorescence meristems and increases the number of reproductive organs, ultimately resulting in increased grain yield [35]. Identifying hormone-responsive genes in the wheat spike may provide important insights for the cloning of specific genes encoding regulators of wheat production.

Pre-harvest sprouting in wheat refers to the germination of seeds in the spikes after physiological maturity, but before harvest, which leads to decreased grain yield and end-use quality as well as considerable economic losses [36]. Pre-harvest sprouting in wheat mainly results from the breaking or lack of seed dormancy under humid conditions [37]. Seed dormancy is primarily regulated by the balance between ABA and GA [38]. However, in *A. thaliana*, IAA also controls seed dormancy via its stimulatory effects on ABA signalling [15]. Accordingly, the application of exogenous IAA enhances the accumulation of ABA in wheat spikes [18]. There is little information available on the effects of endogenous hormones in wheat spikes on pre-harvest sprouting. Related research may benefit from the gene expression data generated in this study.

Drought stress adversely impacts many aspects of plant physiology, especially the photosynthetic capacity, thereby diminishing crop growth and productivity. Drought tolerance is a complex trait controlled by various genes, transcription factors, microRNAs, hormones, proteins, co-factors, ions, and metabolites [39]. In addition to quantitative trait locus mapping, transcriptomic, proteomic, and metabolomic techniques have been used to identify drought signalling pathways in wheat [39–44]. However, there has been relatively little research focused on wheat spikes, which are an important photosynthate source for the grain-filling period [45]. Consequently, the molecular mechanism underlying drought tolerance in wheat spikes remains relatively uncharacterised [28]. Hormone responses and homeostasis are key physiological mechanisms associated with drought stress tolerance [39, 43, 46]. Reddy et al. [47] reported that auxin, ABA, brassinosteroid, CK, ET, GA, and JA are involved in the drought stress response of wheat leaves, and that ABA, auxin, and ET are particularly important for this response. In the current study, we determined that ABA, JA, and auxin (IAA) play a major role in the drought stress response of wheat spikes (Fig. 5b). The accumulated ABA is thought to activate the acclimation and adaptation response that allows longer term survival under drought stress conditions [48, 49]. Additionally, decreases in the auxin content to inhibit growth may also mediate drought stress responses [50]. Our results suggest that ABA signalling is a key factor for improving the drought resistance of wheat spikes, whereas IAA decreases the drought tolerance of wheat spikes. Our data may be useful for clarifying the key molecular mechanism regulating drought responses in wheat spikes during the grain-filling period.

The contribution of phytohormones to the wheat defence mechanisms against FHB remains relatively unknown, although studies have been completed to elucidate the roles of phytohormones related to wheat FHB resistance [19, 20, 23, 51–53]. The infection of wheat heads by *F. graminearum* leads to a significant increase in the accumulation of SA, JA, ABA, and IAA [18]. Additionally, SA signalling is reportedly important for *A. thaliana* and wheat defences against *F. graminearum* [22–24, 54]. Previous studies confirmed that JA signalling is a key factor for improving wheat resistance to FHB [18, 19, 52]. A comparison of the transcriptomic data of FHB-susceptible and -resistant wheat lines revealed a strong correlation between the upregulation of JA signalling and wheat FHB resistance. The application of exogenous JA and the inhibition of JA biosynthesis by BSMV (barley stripe mosaic virus)-VIGS (virus induced gene silencing) can increase and decrease FHB resistance, respectively [18]. In the current study, we confirmed that exogenous MeJA specifically upregulates the expression of numerous genes, many of which are closely related to defence response processes (Table 1). These genes may be important for JA-induced FHB resistance. Exogenous ABA reportedly increases the susceptibility of wheat to *F. graminearum* infections [18, 53]. However, we observed that ABA induced the expression of the highest number of FHB-responsive genes among the seven tested phytohormones, including many detoxification-related genes. A possible mechanism underlying the negative effect of ABA on FHB tolerance involves the suppressed expression of phenylalanine pathway genes due to ABA. Previous studies of *A. thaliana* [55, 56] demonstrated that ABA may suppress lignin production by regulating phenylpropanoid biosynthesis. Secondary cell wall thickening is one of the main mechanisms that prevents the spread of *F. graminearum* in wheat, and is due to the deposition of hydroxycinnamic acid amides, flavonoids, and lignin, which are synthesised via a phenylpropanoid metabolic shunt [57]. Suppression of the phenylalanine pathway involved in flavonoid and lignin biosynthesis may decrease FHB resistance by weakening the physical barriers to the fungus. Additionally, IAA may be crucial for the interaction between wheat and *F. graminearum*. An earlier investigation indicated that *F. graminearum* can produce IAA, thereby contributing to dramatic changes in the IAA contents of infected wheat heads [18]. However, the mechanism by which fungal-derived IAA affects wheat FHB resistance remains unclear. Interestingly, IAA induces the accumulation of ABA [18], which represents a possible explanation. Our transcriptome analysis described herein may promote future research into the role of IAA during *F. graminearum* infections. Moreover, our transcriptional data may provide new insights into the contribution of phytohormones to wheat FHB resistance.

## Conclusions

In this study, we comprehensively analysed the transcriptomic changes in wheat spikes induced by seven phytohormones (IAA, GA, ABA, ET, CK, SA, and MeJA), ultimately resulting in a transcriptional reference map of hormone responses in wheat spikes. We applied this map to investigate the role of hormone signalling pathways in wheat responses to biotic (FHB) and abiotic (water deficit) stresses. The data presented herein may be valuable for elucidating the contribution of phytohormones to wheat spike-related traits.

## Methods

### Plant material and growth conditions

*Triticum aestivum* cv. ‘Roblin’ (provided by Agriculture Canada) plants were grown in climate-controlled chambers under a 16-h day (25 °C):8-h night (20 °C) cycle. The plants were watered as needed and 15–15-15 (N-P-K) fertiliser was applied weekly.

### Hormone treatments

Only flowering heads were used for hormone treatments. Two florets of each fully developed spikelet from a whole spike at the mid-anthesis stage were treated with 10 μl 10% methanol:water solution with or without (control) 2 mM IAA, 1 mM SA, 1 mM MeJA, 0.38 mM ABA, 0.5 mM GA_3_, or 0.5 mM trans-zeatin (a type of cytokinin). Regarding the ET treatment, each head was sealed in a plastic bag with 2 ml freshly prepared 25 mM ethephon (pH = 11). All of the hormone solutions were used to treat florets within 1 h of being prepared. The heads were collected at 24 h after the hormone treatments, and then ground to a fine powder in liquid nitrogen. Each treatment was completed with three biological replicates, each of which comprised at least five heads. No unusual morphological changes were observed in the treated heads. The hormone concentrations of the treatments were based on previous studies [18, 20, 58].

### RNA isolation and microarray and qRT-PCR analyses

For the microarray analysis, total RNA was extracted with the TRIzol reagent (Invitrogen, Shanghai, China). The quality of the extracted RNA was monitored with the ND-1000 spectrophotometer (NanoDrop Technologies, Wilmington, DE, USA) and by agarose gel electrophoresis. The Affymetrix wheat genome array, with 61,127 probe sets representing up to 55,052 transcripts, was used for gene expression profiling at CapitalBio Corporation (Beijing, China). Normalised values are herein presented in the log_2_-transformed form. The normalised data were analysed with the R package SAM (significance analysis of microarrays) [59] to identify candidate genes that were significantly and differentially expressed according to the following criteria: *q*-value < 0.05, fold-change ≥2 in the expression ratio (i.e. log_2_ ratio ≥ 1.0 or ≤ − 1.0; hormone treatment vs control), and signal intensity > 1000 for at least one of the probes for a given gene. A hierarchical clustering analysis of the RNA data involving the average linkage method was used to estimate the global changes between biological replicates and treatments.

Regarding the qRT-PCR analyses, RNA samples were extracted with the TRIzol reagent (Invitrogen). The RNA quality was monitored with the ND-1000 spectrophotometer and by agarose gel electrophoresis before and after a DNase I treatment (Takara, Dalian, China). The RNA was purified with the RNeasy kit (Tiangen, Beijing, China), after which cDNA was synthesised with the PrimeScript RT reagent kit (Takara) and 1 μg total RNA as the template. Primers were designed and qRT-PCR analyses were completed as previously described [60]. Details regarding the qRT-PCR primers are listed in Additional file 1: Table S7. The primers were designed based on the consensus sequences in the NCBI unigene database (http://www.ncbi.nlm.nih.gov). Three housekeeping genes encoding aldehyde oxidase (*AOx*, NCBI UniGene Ta.6172), glyceraldehyde-3-phosphate dehydrogenase (*w-GAPDH*, Ta.66461), and heterogeneous nuclear ribonucleoprotein Q (*hn-RNPQ*, Ta.10105) were amplified as reference genes for the normalisation of the data [20].

### Gene annotation and GO term enrichment analysis

Blast2GO (version 2.8) was used to annotate the gene transcripts with GO terms. For each treatment, all of the enriched GO terms (biological process, level 2) were identified based on a singular enrichment analysis, which was completed with the agriGO tool (http://systemsbiology.cau.edu.cn/agriGOv2/) [61].

## Additional file

Additional file 1: **Table S1.** List of genes differentially expressed in wheat spikes in response to seven hormones. **Table S2.** Gene enrichment analysis of DEGs in response to various hormones. **Table S3.** List of 1599 DEGs responsive to both *F. graminearum* and hormone treatments. **Table S4.** Gene enrichment analysis of DEGs commonly regulated by hormones (ABA, MeJA, or IAA) and *F. graminearum.* **Table. S5.** List of 157 DEGs responsive to water deficit stress in the glume and hormone treatments. **Table S6.** List of 29 genes that exhibited the opposite expression patterns in response to water deficit stress and IAA. **Table S7.** Details regarding the qRT-PCR primers. (XLSX 583 kb)

## Abbreviations

ABA: Abscisic acid
AOx: Aldehyde oxidase
BSMV-VIGS: Barley stripe mosaic virus virus induced gene silencing
CK: Cytokinin
DEG: Differentially expressed gene
ET: Ethylene
FHB: Fusarium head blight
GA: Gibberellic acid
GAPDH: Glyceraldehyde-3-phosphate dehydrogenase
GO: Gene ontology
hn-RNPQ: Heterogeneous nuclear ribonucleoprotein Q
IAA: Indole acetic acid
MeJA: Methyl jasmonic acid
SA: Salicylic acid
SAM: Significance analysis of microarrays
